# *In situ* two-dimensional imaging quick-scanning XAFS with pixel array detector

**DOI:** 10.1107/S0909049511031232

**Published:** 2011-09-16

**Authors:** Hajime Tanida, Hisao Yamashige, Yuki Orikasa, Masatsugu Oishi, Yu Takanashi, Takahiro Fujimoto, Kenji Sato, Daiko Takamatsu, Haruno Murayama, Hajime Arai, Eiichiro Matsubara, Yoshiharu Uchimoto, Zempachi Ogumi

**Affiliations:** a Kyoto University, Japan

**Keywords:** XAFS, two-dimensional imaging, *in situ*, quick scanning, pixel array detector

## Abstract

Quick-scanning X-ray absorption fine structure (XAFS) measurements were performed in transmission mode using a PILATUS 100K pixel array detector (PAD). The method can display a two-dimensional image for a large area of the order of a centimetre with a spatial resolution of 0.2 mm at each energy point in the XAFS spectrum. The time resolution of the quick-scanning method ranged from 10 s to 1 min per spectrum depending on the energy range. The PAD has a wide dynamic range and low noise, so the obtained spectra have a good signal-to-noise ratio.

## Abstract 2

Two-dimensional imaging quick-scanning XAFS measurements were performed using a pixel array detector.

## 1. Introduction

X-ray absorption fine structure (XAFS) is a powerful technique used to investigate the local structure of amorphous and solution samples such as battery materials (McBreen, 2009 ▶). It is important to fully understand the concentration profile and the chemical state of the element in a given material especially in terms of chemical reactions. In this study we developed a two-dimensional X-ray imaging method with a quick-scanning XAFS (QXAFS) measurement for *in situ* analysis of a large sample area. The time-resolved method shows the distribution of elements and their chemical states in the materials during a chemical reaction. In conventional XAFS measurements the energy of the monochromator is constant when acquiring data, and it takes more than a few tens of minutes to obtain a spectrum. In contrast, with the QXAFS method, the data are acquired on-the-fly while driving the monochromator (Frahm, 1988 ▶). It takes only a few minutes to obtain an extended X-ray absorption fine structure (EXAFS) spectrum. Quick-scanning EXAFS methods on the millisecond timescale are possible with a monochromator that uses a piezo-stage table (Richwin *et al.*, 2001 ▶) or galvano-scanner stage (Uruga *et al.*, 2007 ▶). As is well known, dispersive XAFS (DXAFS) is the fastest measurement method (Matsushita & Phizackerley, 1981 ▶). DXAFS is feasible for obtaining transmission imaging data for a large area by scanning the sample, but it generally gives a poorer EXAFS data quality for inhomogeneous samples. These types of imaging data show a distribution of particles in the material and XAFS shows the type of element and the chemical state. X-ray microscopy and tomography combined with the X-ray absorption method is an excellent technique that has been previously studied (Beale *et al.*, 2010 ▶; Grunwaldt & Schroer, 2010 ▶). In addition, a two-dimensional X-ray imaging technique with a microbeam and translation stage has been reported on battery samples (Nakai, 1998 ▶). A charge-coupled-device (CCD) detector with a high spatial resolution of a few micrometres is usually used for the imaging measurement, but such a detector has a smaller detection area, lower dynamic range and higher background than a pixel array detector (PAD). In this study a PILATUS 100K detector from DECTRIS (Broennimann *et al.*, 2006 ▶), Switzerland, was used as the PAD. It has a spatial resolution of about 0.2 mm and an effective area of about 8 cm wide and 3 cm high. Therefore, the high sensitivity and wide dynamic range of the data obtained by the PAD provide high-quality XAFS spectra in just a few minutes of time resolution. It is crucial that the results are obtained within a few minutes with *in situ* measurements in order to properly observe changes in chemical state distribution as well as microsecond reactions. In this study, *in situ* two-dimensional imaging XAFS of a battery sample is performed.

## 2. Experimental

The data were recorded at beamline BL01B1 at SPring-8, Hyogo, Japan (Uruga *et al.*, 2009 ▶). The size of an X-ray beam from a bending-magnet beamline is larger than that from an undulator beamline. The X-ray beam size was around 0.2 mm high and 10 mm wide on the sample. A Si (111) crystal was used as the monochromator crystal. Two Rh-coated mirrors were used for removing higher harmonics. The first mirror, in front of the monochromator, collimated the X-ray beam to improve the energy resolution, and the second mirror, behind the monochromator, vertically focused the X-ray beam to around 0.2 mm. The monochromator at BL01B1 is of the SPring-8 standard two-crystal fixed-exit type, and has two stages: one for the rotation of crystals and another for translation of the first crystal (Yabashi *et al.*, 1999 ▶). The translation stage of the first crystal is moved to the position calculated from the monochromator angle and therefore the X-ray beam on the sample is vertically stable. For quick-scanning measurements the first-crystal position was fixed at the centre of the scan range and only the rotation stage of the monochromator was moved continuously. The beam position moved less than 0.001 mm because the X-ray beam was focused by the second mirror. The monochromator angle was monitored on-the-fly by a rotary encoder and an IK220 PCI board (Heidenhain, Germany).

The intensity of the incident X-rays was measured in a 17 cm-long ionization chamber. The current from the ionization chamber was measured as a voltage value using a Keithley 428 current amplifier (from Keithley, USA), and the voltage value was converted to a frequency value using a DS-VFC2 voltage-to-frequency converter (Seiko EG&G, USA). The rise time of the current amplifier was set to the minimum value. The pulse value from the voltage-to-frequency converter was counted on-the-fly by a scaler that had a data storage memory. The scaler was an Axcpci3901 high-speed counter timer from ARCUS, Japan, which generated a timing gate signal, while the IK220 encoder board counted the encoder value synchronized by the gate signal.

**Figure 1:**
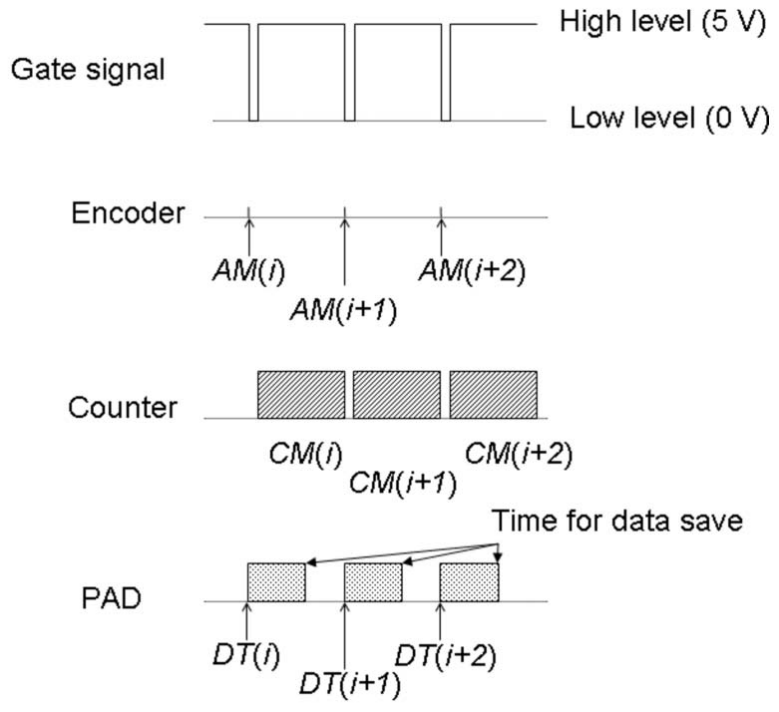
Timing diagram of the two-dimensional imaging QXAFS measurement.

A PILATUS 100K detector from DECTRIS, Switzerland, was used as the PAD. The image was recorded with a timing signal. It took around 0.02 ms to save the data when using the encoder board and the counter, and 3 ms when using the PAD because the image data saved was large, a few hundred kilobytes. Fig. 1 ▶ shows a schematic diagram of the timing diagram for acquiring the data. The gate signal gap is more than 3 ms. The encoder angle *AM*(*i*) is recorded to the memory when the high level of the gate signal (5 V) goes to a low level (0 V). The angle data of the spectrum is represented by half the sum of the angles before and after the gate signal, [*AM*(*i*) + *AM*(*i* + 1)]/2, when the counter takes the data at *CM*(*i*). The gap of 0.02 ms is negligible as a constant dead-time. The PAD data are recorded when the gate signal goes from high to low and the counter starts at *DT*(*i*). The interval of the data acquisition is more than 3 ms. The XAFS spectra of the PAD image are normalized by blank images obtained without a sample. The best normalization method would use a blank image at each energy point. However, this method is difficult to combine with the quick-scanning method in this study. The top-off mode operation of the storage ring minimizes normalization effects.

## 3. Result and discussion

**Figure 2:**
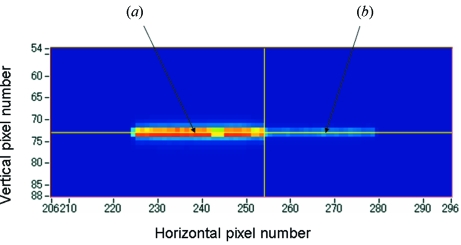
PAD image of a LCO electrode. The vertical and horizontal scales correspond to the PAD pixel number, 0.172 mm. The right-hand side is covered by a Li anode. XAFS spectra in Fig. 3 ▶ are obtained using pixels (*a*) and (*b*).

**Figure 3:**
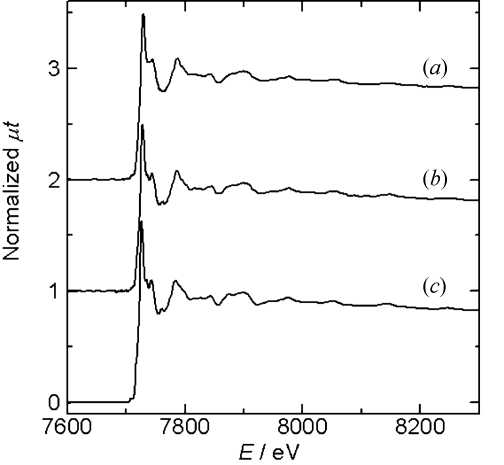
Co *K*-edge XAFS spectra of the LCO electrode. In (*b*) the electrode is covered by an anode. The two spectra (*a*) and (*b*) are obtained from the pixel regions shown in Fig. 2 ▶. Spectrum (*c*) is measured by a conventional transmission method. The spectra have been vertically offset for comparison.

**Figure 4:**
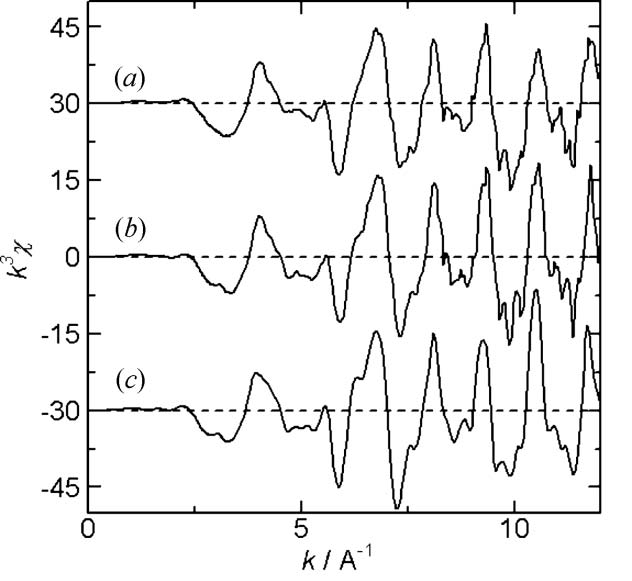
Co *K*-edge EXAFS spectra of the LCO electrode. In (*b*) the electrode is covered by an anode. The two spectra (*a*) and (*b*) are obtained from the pixel regions shown in Fig. 2 ▶. Spectrum (*c*) is measured by a conventional transmission method. The spectra have been vertically offset for comparison.

Fig. 2 ▶ shows an image of the PAD. The sample is a lithium cobalt oxide (LCO) cathode electrode. The incident X-ray energy was about 8 keV above the Co *K*-edge. The aperture of the slit in front of the ionization chamber and sample was about 0.2 mm high and 10 mm wide. The vertical and horizontal scales correspond to the pixel number of the PAD, which is 0.172 mm. The PAD image in Fig. 2 ▶ was observed to be larger than the aperture size of the slit because the X-ray beam diffused and the PAD was set after the sample. The right-hand side of the transmission image in Fig. 2 ▶, part (*b*), was covered with a lithium anode and a Ni film, where a voltage for the charge–discharge was applied, and the X-ray intensity on the detector is lower than on the left-hand side. The normalized XAFS spectra of the other pixels are shown in Fig. 3 ▶. Spectrum (*c*) in Fig. 3 ▶, measured by a conventional transmission method with an ionization chamber, is shown for comparison. The background subtraction and normalization was performed using the data analysis software *REX2000* from Rigaku, Japan (Taguchi *et al.*, 2002 ▶). The spectra were normalized by the pixel value of the blank image, which was measured without a sample. The count rate of the detector was adjusted by using an aluminium foil attenuator, and limited to about 1 Mcount s^−1^. The count rate of the detector was previously checked and the detector had no dead-time at 1 Mcount s^−1^. The count rate was sufficient for 10 ms at each energy point for obtaining spectra. A QXAFS spectrum was measured in 30 s. As a battery reaction mostly takes place over several tens of minutes to several hours, this time resolution is suitable for observing the changes occurring in the sample. The observation area and the spatial resolution of the detector were suitable for observing the chemical reaction of the cathode in the presence of the anode. The spectra were obtained with a low background and a good signal-to-noise ratio, and therefore sufficient for further Fourier transform and curve-fitting analysis of the EXAFS spectrum, as shown in Figs. 4 ▶ and 5 ▶. The Fourier transforms were performed on *k* ^3^-weighted EXAFS spectra from 3.5 to 12 Å^−1^. The sample information and curve-fitting analysis will be described elsewhere in detail in a future work.

## 4. Conclusion

*In situ* transmission two-dimensional imaging QXAFS using a PAD was successfully demonstrated. The method is widely applicable to the field of materials science for solutions, amorphous materials and batteries. The size of the battery electrode sample, for example, is of the order of a few centimetres, so it is necessary to observe the distribution of the electrochemical reaction in such an area. This method is suitable for large samples of the order of a few centimetres with a large dynamic range and low background data. It can deliver the optimal time and space resolutions. If a resolution of a few micrometres for a small sample is needed, a CCD detector can be used; it is flexible in terms of the type of detector it uses.

## Figures and Tables

**Figure 5:**
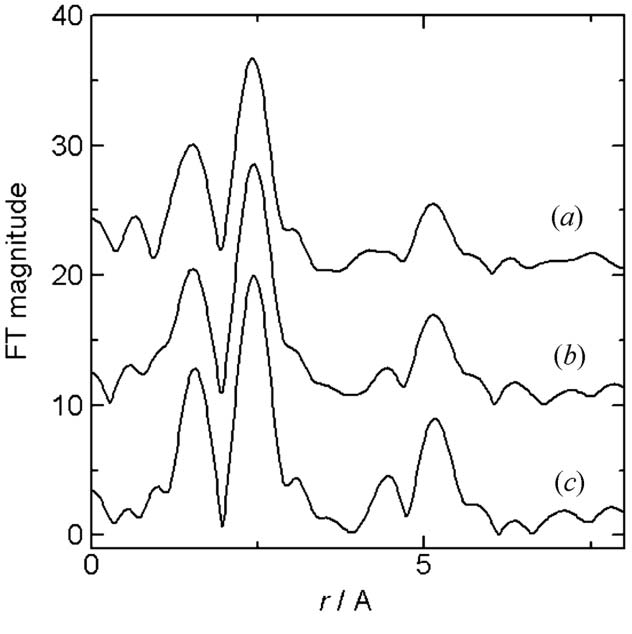
Fourier transforms of the Co *K*-edge EXAFS spectra of the LCO electrode. In (*b*) the electrode is covered by an anode. The two spectra (*a*) and (*b*) are obtained from the pixel regions shown in Fig. 2 ▶. Spectrum (*c*) is measured by a conventional transmission method. The spectra have been vertically offset for comparison.
